# Investigation of Vaccine-Induced Immune Response After Vaccination Against Respiratory Viruses in Immunocompromised Patients With or Without Hemato-Oncological Diseases (RESPONSE): Protocol for a Prospective Cohort Study

**DOI:** 10.2196/88520

**Published:** 2026-07-23

**Authors:** Eloísa Felipe Fumero, Jannik Stemler, Rosanne Sprute, Katja M Sauer, Henning Gruell, Christoph Kreer, Florian Klein, Sebastian J Theobald, Jan Rybniker, Oliver A Cornely, Lara Kappes, Louise M Cremer, Sibylle C Mellinghoff

**Affiliations:** 1 Department I of Internal Medicine, Center for Integrated Oncology Aachen Bonn Cologne Duesseldorf (CIO ABCD) and Excellence Center for Medical Mycology (ECMM) University Hospital Cologne Cologne Germany; 2 Institute of Translational Research, Cologne Excellence Cluster on Cellular Stress Responses in Aging-Associated Diseases (CECAD) University of Cologne Cologne Germany; 3 German Center for Infection Research (DZIF) Partner Site Bonn-Cologne Cologne Germany; 4 Institute of Virology, Faculty of Medicine and University Hospital Cologne University of Cologne Cologne Germany; 5 Clinical Trials Centre Cologne (ZKS Köln) University of Cologne Cologne Germany

**Keywords:** vaccination, immunocompromised host, hematological patients, humoral immune response, cellular immune response, respiratory viral infections

## Abstract

**Background:**

Infections with respiratory viruses such as SARS-CoV-2 and influenza are significant international public health concerns. While patients with cancer remain the most vulnerable group, they show poor vaccine response in general. Immunological data in this population are limited and mainly focus on serological parameters. However, in these patients, cellular, and especially T-cell, responses often seem to be induced more reliably than humoral responses.

**Objective:**

To gain further insights into vaccine-induced immunity, the RESPONSE study will analyze the effect of early and late booster vaccination on humoral and cellular responses, with special focus on T cell–induced immune responses. In addition, we aim to investigate factors influencing humoral and cellular vaccine-induced immunity in patients with hematological and oncological malignancies, including state of disease, treatment, and demographic factors.

**Methods:**

Humoral immune responses will be assessed by measuring binding and neutralizing antibodies using standardized assays. Cellular immunity will be evaluated using functional assays such as flow cytometry and FluoroSpot, as well as in-depth analyses using additional exploratory assays as appropriate. Immune responses will be correlated with clinical parameters, including disease status, treatment, and demographic factors.

**Results:**

This study was initiated following ethics approval and is currently recruiting participants. Enrollment commenced on March 25, 2025, and is ongoing, whereas biosample collection and follow-up visits are nearing completion for most participants. Final data cleaning, dataset integration, and statistical analyses of adaptive immune responses are planned from the third quarter of 2026 onward.

**Conclusions:**

This study intends to lay a foundation for a structured translational research platform on vaccination to aim for best protection from infection by different respiratory pathogens. Long-term objectives are reaching best possible protection from vaccine-preventable disease with a first focus on influenza infection. In addition, we plan to investigate vaccine-induced immune responses to the recently approved respiratory syncytial virus vaccine using this platform and possibly extend this to further vaccines in the future. Urgent questions, such as the influence of different targeted therapies on vaccine immune response, will be part of these projects.

**Trial Registration:**

ClinicalTrials.gov NCT06612515; https://clinicaltrials.gov/study/NCT06612515

**International Registered Report Identifier (IRRID):**

DERR1-10.2196/88520

## Introduction

Respiratory virus infections are a significant cause of morbidity and mortality, particularly among immunocompromised patients. These infections are caused by a variety of viral pathogens, including influenza viruses, respiratory syncytial virus (RSV), parainfluenza viruses, adenoviruses, rhinoviruses, and coronaviruses, among others. In the general population, these infections typically result in self-limiting illnesses. However, in individuals with compromised immune systems, such as those undergoing chemotherapy, organ transplant recipients, patients with hematological malignancies, and those with HIV, the consequences can be severe and often life-threatening. For example, patients with hematological and oncological diseases are at elevated risk of severe morbidity and mortality from influenza infections. Compared to influenza-associated hospitalization rates in the general population (37.9 per 100,000 person-years for persons aged 50 to 64 years) [1], patients with cancer are hospitalized at a rate of 219 per 100,000 person-years for patients younger than 65 years [2].

The immune system plays a crucial role in defending against viral pathogens. Immunocompromised patients, due to either primary immune deficiencies or secondary immunosuppression (eg, from immunosuppressive therapies), show impaired immune responses that hinder the effective clearance of viral infections. This impairment can lead to prolonged viral shedding; increased risk of secondary bacterial infections; and more severe disease manifestations, including acute respiratory distress syndrome and multi-organ failure. The management of respiratory virus infections in immunocompromised patients requires a multidisciplinary approach. Prophylactic measures, including vaccination and the use of antiviral prophylaxis, are crucial in reducing the incidence of these infections [3-5].

Given the significant impact of respiratory virus infections on immunocompromised patients, ongoing research is crucial to improve preventive strategies. This research includes studies on viral pathophysiology, host immune responses, and the development of optimal vaccination schedules. The emergence of new viral pathogens such as SARS-CoV-2 underscores the importance of surveillance and preparedness in this vulnerable population [6,7].

Respiratory virus infections represent a major health concern for immunocompromised patients, necessitating comprehensive and specialized care [2,3,8]. Continued advancements in diagnostics, therapeutics, and preventive measures are vital to mitigate the impact of these infections and improve the quality of life and survival of immunocompromised individuals.

The management of respiratory virus infections in immunocompromised patients requires a multidisciplinary approach and prophylactic measures, including vaccination, to reduce the incidence and severity of these infections. However, the efficacy of vaccination is often suboptimal in immunocompromised individuals and not well assessed in clinical trials [9-13].

A poor humoral immunogenicity has been reported for most available vaccines in hematological patients [12,14-18]. In particular, the affection of the B-cell axis by disease, treatment, or both impacts humoral vaccine immune response [19].

The humoral vaccine-induced immune response facilitates early protection directly after vaccination, whereas long-term protection warrants antibody persistence as well as immune memory cells [20]. Data from immunocompetent persons suggest that the cell-mediated immune response may be a better correlate of protection against virus infections in vaccinated adult patients with poor immune responses [21,22] than humoral immunity due to its major role in recovery from infection and virus clearance [23].

Immunological data in patients with hematological malignancies are generally scarce and mainly focus on serological parameters. However, in these patients, cellular immunity and especially T-cell responses often seem to be induced more reliably than humoral responses, as observed in other immunocompromised populations [24] and for other vaccines [24,25].

To close this knowledge gap, we will perform an observational study to prospectively analyze the effect of early and late booster vaccination, with special focus on virus-specific T-cell responses. Samples will be collected from patients who have been vaccinated as part of clinical routine.

## Methods

### Study Design

The RESPONSE study is a prospective monocentric noninterventional study of vaccine efficacy in immunocompromised patients. The study was registered on ClinicalTrials.gov on September 19, 2024. All immunocompromised patients who are vaccinated or willing to be vaccinated against respiratory viruses in accordance with Robert Koch Institute guidelines will be eligible. Eligibility depends on agreement by signing an informed consent form (ICF). A total of 1000 patients are planned to be enrolled.

Participating departments will screen all immunocompromised patients for eligibility according to the following definition: for immunocompromised states, the classification by Wiedermann et al [26], the “stages of immunosuppression,” will be considered, and patients in stages 1 to 3 will be eligible for study inclusion.

**Figure 1:**
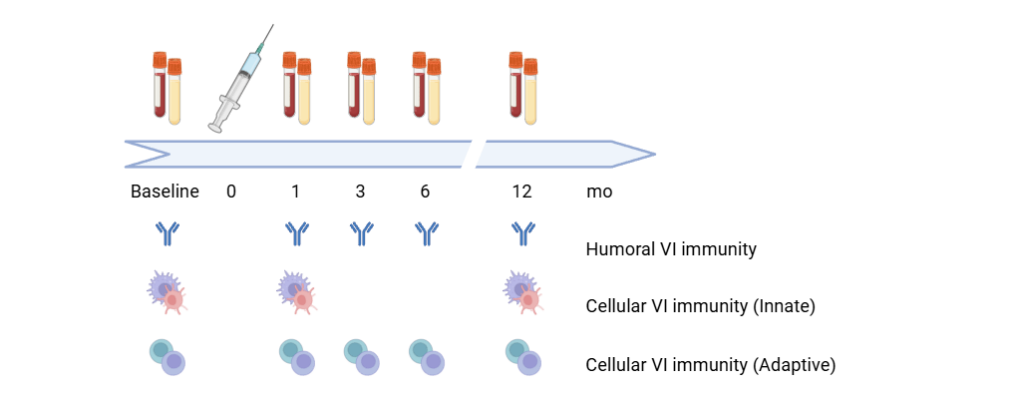
Sampling for analyses of vaccine-induced (VI) humoral and cellular immunity.

Eligible patients will be approached and informed about the study by their physicians. Patients will receive both oral and written study information and will be given sufficient time to ask questions and consider participation prior to enrollment. If a patient agrees to participate and signs the ICF, basic data regarding vaccination history and underlying disease, among other variables, will be collected. Samples for the assessment of vaccine-induced immune response will be drawn at baseline and 1, 3, 6, and 12 months after vaccination (−7 days to +7 days). Study personnel will coordinate sample assessment schedules and inform the participating physicians of upcoming blood samplings. The samples will be taken on planned physician appointments together with regular laboratory assessment due to the underlying disease. Study-related blood sampling will be performed at the same time points and from the same vein puncture as for routine standard procedures. An amount of 30 to 40 mL will be obtained (1 serum and 2-3 ethylenediaminetetraacetic acid [EDTA] tubes, as shown in Figure 1). No additional interventions (eg, vein puncture) or treatments are foreseen as part of study participation.

Blood samples will be stored pseudonymized and frozen at University Hospital Cologne (Translational Research for Infectious Diseases and Oncology [TRIO] building).

All therapies and diagnostics, including vaccinations, will be administered solely as part of clinical routine and as recommended by appropriate guidelines (eg, recommendations from the German Standing Committee on Vaccination [STIKO] by the Robert Koch Institute). The German Arzneimittelgesetz (Medicinal Products Act), which regulates interventional clinical trials involving medicinal products, hence does not apply to this noninterventional observational study, and participating physicians, therefore, do not require Good Clinical Practice (GCP) certification.

### Objectives

The study objectives are as follows: (1) to compare antibody titers between different subgroups of immunocompromised patients after vaccination against respiratory viruses, (2) to compare antibody response rates between different subgroups of immunocompromised patients after vaccination against respiratory viruses, (3) to analyze T-cell response after vaccination against respiratory viruses, (4) to investigate factors (such as age, sex, treatment, and underlying disease) influencing the humoral and cellular vaccine-induced immune response in patients vaccinated against respiratory viruses, and (5) to improve vaccination strategies to achieve better protection against vaccine-preventable diseases caused by different respiratory pathogens in high-risk populations.

### Data Collection

Every study participant will be given a pseudonym. Study personnel will capture the following data at study entry:

- Patient characteristics—sex (male or female) and age (years)
- Details on vaccination against respiratory viruses—date and type of vaccination
- Further vaccination history
- Details of the underlying disease and its treatment
- Data on infections, clinical signs and symptoms, identified pathogens, hospitalization, and severity of infection, as well as outcome within the interval since the last study sample

Longitudinal clinical and immunological follow-up data will be collected during predefined study visits after vaccination. Humoral immune responses will be assessed via measurement of binding and neutralizing antibodies using standardized serological assays, whereas cellular immune responses will be evaluated using functional T-cell assays, including flow cytometry–based analyses and FluoroSpot assays. Immune response data will be correlated with clinical characteristics, including underlying disease, treatment regimen, vaccination timing, prior infections, and demographic variables, to identify factors associated with impaired or enhanced vaccine-induced immunity. Due to the exploratory and observational nature of the study, additional immunological analyses may be performed as scientifically appropriate to further characterize vaccine-induced immune responses in this high-risk population.

Data will be pseudonymized for documentation. Access to personal data by third parties is not possible. Nonpersonal data (age, gender, illness, and medication) are also recorded using a survey form linked to the pseudonymization number. Personal data (name and date of birth) are handwritten in a pseudonymization list alongside the pseudonymization number. The pseudonymization list, the survey form, and consent forms are securely stored in their original form at the TRIO building. All data and information are subject to the legal provisions of the Federal Data Protection Act, the European General Data Protection Regulation, and confidentiality obligations. Participants have the right to obtain information about personal data that are collected and processed as part of the study at any time. They also have the right to receive a free copy of their data. Furthermore, participants have the right to have incorrect personal data corrected, have their data deleted, or restrict the use of their data.

### Study Procedure

#### Site Specifications

In principle, all departments of University Hospital Cologne will be eligible to participate in this study. Informed consent will be obtained by treating physicians.

#### Sample Size

A sample size of 1000 participants is planned. Due to an observational cohort design, a formal sample size calculation is not applicable. This is due to the nature of the study, which focuses on observing and analyzing real-world data without predefined experimental conditions. As such, the study relies on available cohorts rather than a controlled intervention, and the sample size is determined by the population under observation rather than a statistical power analysis.

### Eligibility

#### Inclusion Criteria

Inclusion criteria are as follows: (1) signed ICF, (2) patients with immunosuppression either from treatment or underlying diseases, (3) adult patients who are vaccinated or willing to be vaccinated against respiratory viruses in accordance with current STIKO recommendations, and (4) application of the vaccine in accordance with the Summary of Product Characteristics and relevant guidelines.

#### Exclusion Criteria

The exclusion criterion is patients unwilling or ineligible for vaccination under current STIKO recommendations.

#### Participant Withdrawal Criteria

A participant may withdraw from the clinical trial at any time for any reason without consequences. Withdrawal from the study is permanent: once a participant withdraws, they shall not be allowed to enter the study again. Withdrawal from the study will have no influence on the availability of vaccination for that patient.

In case of premature withdrawal, the reason should be collected, but this is not obligatory.

In case of withdrawal, all blood samples will be destroyed according to data protection requirements. Data cannot be removed from already completed scientific analyses. A participant who withdraws from the trial will not be replaced.

### Enrollment

Eligible study participants will be identified by their treating physicians. If the participant satisfies the eligibility criteria and signs the ICF, they will be enrolled in the study by entering their data into the enrollment log. Basic data regarding vaccination history and underlying disease, among other variables, will be captured (as outlined in the Data Collection section).

### Study Conduct

#### Titer Assessment

Blood samples for analysis of vaccine-induced immune response will be drawn at baseline and 1, 3, 6, and 12 months after vaccination (−7 days to +7 days).

Study personnel will coordinate titer assessment schedules and inform participating physicians of upcoming blood samplings. The samples will be taken on planned physician appointments together with regular laboratory assessment due to the underlying disease. Samples will be analyzed after the last follow-up of each patient.

#### Immunological Assays

Immunological assays focus on (1) evaluation of pathogen-specific binding and neutralizing antibodies using in-house enzyme-linked immunosorbent assay (ELISA) and virus neutralization tests, including subclassification of antibodies and evaluation of neutralizing and nonneutralizing antibody functions; (2) evaluation of potential cross-reactivity of the induced antibodies with other human pathogens using immunological methods (eg, in-house ELISA); (3) evaluation of the innate immune response to vaccine candidates, for example, by determining cell populations via flow cytometry on a BD FACSymphony A3 Cell Analyzer; (4) evaluation of the cellular immune response, including (memory) B and T cells and T helper cell groups, for example, using immunological methods such as FluoroSpot (using the Mabtech FluoroSpot Plus kit), as well as via fluorescence-activated cell sorting analysis; and (5) identification of potential biomarkers of a specific immune response.

### Management of Samples

#### Collection and Preparation

For all participants, blood will be collected in one 9-mL serum tube and three 9-mL EDTA tubes.

Immediately prior to the blood draw, the staff member performing the procedure will verify the participant’s identity.

Labels will be provided and attached to the tubes. The tubes will be transferred to the laboratory within 4 hours and processed within 12 hours. The sample will be used for antibody titer and T-cell immunity analysis.

Participant number, date of sampling, number of aliquots, date and time of preparation, and the participant’s consent are documented in a sample identification list and recorded as source data.

#### Storage

Samples will be stored at the TRIO laboratory in Cologne in ultralow-temperature freezers (−80 and −150 °C).

### Statistical Analysis and Data Management

#### Overview

Descriptive statistics will be used to summarize demographic, clinical, and immunological variables. For metric variables, the arithmetic mean, SD, coefficient of variation, minimum, maximum, median, and IQR will be reported as appropriate. For log-normally distributed parameters, geometric means and geometric coefficients of variation will additionally be provided. Categorical variables will be summarized using frequencies and percentages. The primary study outcomes are the magnitude and dynamics of humoral and cellular immune responses following vaccination and booster vaccination in patients with hematological and oncological malignancies. Humoral immune responses will be assessed by quantitatively binding antibody titers and neutralizing antibody levels. Cellular immune responses will be evaluated using flow cytometry–based assays, FluoroSpot analyses, and additional exploratory immunological assays where appropriate. Secondary outcomes include comparisons of immune responses between early and late booster vaccination groups, evaluation of responder and nonresponder rates, and identification of demographic and clinical factors associated with vaccine-induced immunity. Immune response analyses will include predefined subgroup comparisons according to underlying malignancy, treatment modality, disease status, age, sex, and relevant comorbidities. Longitudinal analyses of repeated measurements obtained at baseline and follow-up visits (eg, 1, 3, 6, and 12 months after vaccination or booster vaccination) will be performed using mixed-effects regression models or generalized estimating equations, as appropriate, to account for within-subject correlations over time. Depending on data distribution and end point characteristics, group comparisons will be conducted using Student *t* tests, Mann-Whitney *U* tests, chi-square tests, ANOVA, or regression-based approaches. Multivariable regression analyses will be used to identify independent predictors of humoral and cellular immune responses. Covariates considered for inclusion may comprise age, sex, malignancy subtype, disease activity, treatment regimen, and timing of booster vaccination. Results will be reported with corresponding effect estimates and 95% CIs.

Missing data will be assessed regarding extent and pattern. If appropriate, missing values will be handled using multiple imputation or mixed-model approaches that allow for analysis under missing at random assumptions. Sensitivity analyses will be performed to evaluate the robustness of the findings. Due to the exploratory nature of several immunological analyses, multiplicity adjustment methods may be applied where appropriate to control for type I error.

All statistical analyses will be conducted using validated statistical software. Statistical significance will generally be defined as a 2-sided *P* value below .05.

#### Data Management Plan

Data will be recorded securely and electronically. The data are the sole property of the sponsor and must not be made available in any form to third parties, except for authorized sponsor representatives or appropriate regulatory authorities, without written permission from the sponsor. The investigator will ensure that all data are entered legibly, completely, and accurately and conform to source documents.

The investigator will review and approve the data, with their validation serving as attestation of the investigator’s responsibility for ensuring that all data are complete, accurate, and authentic. All information obtained during the study will be recorded digitally in conformity with applicable laws and regulations.

### Ethical Considerations

The study will be performed in accordance with all applicable laws and regulations, including the International Council for Harmonisation of Technical Requirements for Registration of Pharmaceuticals for Human Use for GCP, whose ethical principles originate in the Declaration of Helsinki (current official version: Fortaleza, 2013 [27]), and applicable privacy laws. The Ethics Committee of the University of Cologne approved this study (24-1312_2-NIS).

#### Ethical Standards

GCP requires that, prior to study onset, the protocol and any other written information regarding this study to be provided to the participants must be approved by an institutional review board (IRB) or independent ethics committee (IEC).

The investigator agrees to allow the IEC or IRB direct access to all relevant documents.

The IEC or IRB must be constituted in accordance with all applicable regulatory requirements. All approvals should be signed by the IEC or IRB chairman or designee and must identify the IEC or IRB name and address, the clinical protocol by title and/or protocol number, the documents received and their version number, and the date when approval and/or positive opinion was granted.

The sponsor will provide the investigator with relevant documents that are needed for IEC or IRB review and approval of the study. The sponsor must receive copies of the IEC or IRB approval and any other information that the IEC or IRB has approved for presentation to potential participants.

If the protocol or any other information that the IEC or IRB has approved is amended during the study, the investigator is responsible for ensuring that the IEC or IRB reviews and approves, where applicable, these amended documents. Copies of the IEC or IRB approval of the amended documents and these amended documents must be forwarded to the sponsor.

#### Data Confidentiality

The study protocol, documentation, data, and all other information generated in this study will be maintained in a secure manner and kept confidential as required by law. An evaluation will be carried out by an external data protection officer. The sponsor will affirm and uphold the principle of the participants’ right to protection against the invasion of privacy.

The investigator will respect and protect the confidentiality of the participants in all possible ways.

Data access and entry will be limited to study personnel.

All information regarding the study, including conduct and results, is confidential. No information can be divulged without written consent from the sponsor.

#### Data Handling and Recordkeeping

A study folder will archive the protocol, correspondence, and other study-related documents at the TRIO building, University Hospital Cologne. Microsoft Excel will be used for documentation of pseudonymized patient data.

All documentation pertaining to the study will be kept by the sponsor for at least 15 years after the end or premature termination of the study.

### Confidentiality, Ownership of Data, and Publication Policy

All information disclosed or provided by the sponsor (or any company or institution acting on their behalf) or produced during the study, including but not limited to the protocol and the results obtained in the course of the study, is confidential prior to the publication of results.

The investigator and any person under their authority agree to keep confidential and not disclose the information to any third party without the prior written approval of the sponsor. The investigator’s collaborators shall be bound by the same obligations as the investigator. The investigator shall inform collaborators of the confidential nature of the study. The investigator and collaborators shall use the information solely for the purposes of the study to the exclusion of any use for their own or for a third party’s account. However, the submission of this protocol and other necessary documentation to the IEC or IRB and the regulatory authority is expressly permitted, with their members having the same obligation of confidentiality. The statistical analysis and final report will result in a published article.

Those investigators who have made the greatest contribution to the generation of data through enrollment of evaluable patients will be taken into account as publication authors. Authors of publications must meet the International Committee of Medical Journal Editors guidelines for authorship and must satisfy the following three criteria: (1) authors must make substantial contributions to the conception and design of the trial, acquisition of data, or analysis of data and interpretation of results; (2) authors must draft the publication or, during draft review, provide contributions (data analysis, data interpretation, or other important intellectual content) leading to significant revision of the manuscript with agreement by the other authors; and (3) authors must provide written approval of the final draft version of the publication prior to submission.

All contributors who do not meet all 3 criteria for authorship will be listed as contributors in an acknowledgment section within the publication, if allowed by the journal, per the International Committee of Medical Journal Editors guidelines for acknowledgments.

## Results

The study was initiated following ethics approval and funded from June 2024 onwards. Enrollment of patients with hematological and oncological malignancies commenced on March 25, 2025, and is ongoing, with longitudinal biosample collection and scheduled follow-up visits nearing completion for most participants currently included in the cohort. To date, a total of 157 patients have been enrolled. The study is estimated to conclude on December 31, 2029. In accordance with the protocol, biological samples and associated clinical data are being collected at predefined time points following vaccination and booster vaccination against respiratory pathogens. Preliminary data on humoral and cellular responses in a small cohort of 17 patients with chronic lymphocytic leukemia (CLL) have shown that, even in the absence of a humoral response, some patients are capable of mounting robust cellular responses following vaccination against respiratory viruses. These results highlight the heterogeneity of vaccine-induced immunity in immunocompromised patients and underscore the need for further research into correlates of protection, the durability of immune responses, and optimal booster strategies for this highly vulnerable population. These preliminary findings are expected to be published in late 2026.

## Discussion

### Expected Findings

The pilot data generated from a small cohort of 17 patients with CLL provides an important first insight into the feasibility and relevance of systematically assessing vaccine-induced humoral and cellular immunity in individuals with hematological malignancies [12,14-19].

Building on these initial observations, the overarching aim of the project is to establish a structured translational research platform that will enable the comprehensive evaluation of immune responses to a variety of vaccines against respiratory pathogens in immunocompromised patients. With enrollment initiated following ethics approval in the first quarter of 2025 and recruitment currently ongoing, the study is positioned to expand its analyses beyond influenza vaccination and include patients across different disease stages, treatment regimens, and demographic backgrounds.

In the long term, this platform seeks to improve protective immunity in vulnerable patient populations by identifying the factors that influence vaccine responsiveness and understanding how timing, booster strategies, and targeted cancer therapies modulate both humoral and cellular immunity. Insights gained from this platform may ultimately inform optimized booster schedules, personalized vaccination strategies, and potential revisions of existing vaccination guidelines for immunocompromised patients. The extension of this research to newly approved vaccines—such as the RSV vaccine—and additional vaccines in the future underscores the intention to create a sustainable, adaptable framework. By integrating immunological monitoring, clinical outcomes, and therapy-specific effects, this platform aims to contribute to evidence-based recommendations that ensure the best possible protection against vaccine-preventable respiratory diseases in high-risk populations.

Previous studies have already demonstrated impaired vaccine-induced immune responses in patients with hematological malignancies, particularly in individuals receiving B cell–depleting therapies or targeted treatments [13]. However, available data remain heterogeneous, often focus predominantly on humoral immunity, and are frequently limited to specific pathogens or single vaccine platforms. A major strength of the present study is the longitudinal and translational design, allowing for simultaneous assessment of humoral and cellular immune responses across a broad spectrum of immunocompromised patients under real-world clinical conditions. At the same time, the observational nature of the study and the heterogeneity of the included patient population may limit direct comparability between subgroups and may introduce potential confounding factors related to disease characteristics and treatment regimens.

The findings generated within this platform will be disseminated through peer-reviewed publications, presentations at national and international scientific conferences, and integration into collaborative discussions with clinical and immunological expert groups. In addition, the generated biobank and longitudinal dataset may constitute a valuable resource for future translational studies investigating correlates of protection, durability of immune responses, and the impact of emerging respiratory pathogens on vulnerable populations.

### Limitations

A key limitation of the current project is the heterogeneity of the study population as patients differ in underlying malignancies, disease status, and ongoing treatments, all of which may influence vaccine-induced immunity. In addition, some diagnostic subgroups—such as the pilot CLL cohort—comprise relatively small sample sizes, limiting the statistical robustness of subgroup analyses. To mitigate these challenges, we aim to increase recruitment across all relevant disease entities, apply standardized sampling and laboratory procedures, and perform stratified analyses to account for known confounders. As recruitment progresses, larger and more balanced subcohorts will strengthen the interpretability and generalizability of the findings.

### Conclusions

This project establishes the foundation for a structured translational research platform dedicated to investigating vaccine-induced immunity in immunocompromised patients. Early pilot data demonstrate the feasibility of longitudinally assessing both humoral and cellular immune responses in patients with hematological malignancies and support further expansion of the platform to additional patient populations and vaccines, including RSV vaccination. The observational data generated within this framework may contribute to a better understanding of factors associated with vaccine responsiveness, including disease characteristics, treatment regimens, and booster timing. In the longer term, these findings may help guide future studies and potentially contribute to the optimization of vaccination approaches and clinical recommendations for immunocompromised populations.

## Data Availability

As this manuscript describes a study protocol for an ongoing observational cohort study, no final datasets are currently available. Following study completion and publication of primary analyses, pseudonymized data may be made available from the corresponding author on reasonable scientific request and in accordance with applicable ethical, institutional, and data protection regulations.

## Abbreviations

CLL: chronic lymphocytic leukemia
EDTA: ethylenediaminetetraacetic acid
ELISA: enzyme-linked immunosorbent assay
GCP: Good Clinical Practice
ICF: informed consent form
IEC: independent ethics committee
IRB: institutional review board
RSV: respiratory syncytial virus
STIKO: German Standing Committee on Vaccination
TRIO: Translational Research for Infectious Diseases and Oncology
